# Trade-Off Analysis of Hardware Architectures for Channel-Quality Classification Models

**DOI:** 10.3390/s22072497

**Published:** 2022-03-24

**Authors:** Alan Torres-Alvarado, Luis Alberto Morales-Rosales, Ignacio Algredo-Badillo, Francisco López-Huerta, Mariana Lobato-Baez, Juan Carlos López-Pimentel

**Affiliations:** 1 Instituto Nacional de Astrofísica, Óptica y Electrónica, Puebla 72840, Mexico; torresalv@inaoep.mx; 2 Facultad de Ingeniería Civil, CONACYT-Universidad Michoacana de San Nicolás de Hidalgo, Morelia 58030, Mexico; 3 Facultad de Ingeniería de la Construcción y el Hábitat, Universidad Veracruzana, Boca del Río, Veracruz 94294, Mexico; frlopez@uv.mx; 4 Higher Technological Institute of Libres, Libres, Puebla 73780, Mexico; mariana.lobato@upaep.edu.mx; 5 Facultad de Ingeniería, Universidad Panamericana, Álvaro del Portillo 49, Mexico City 45010, Mexico; clopezp@up.edu.mx

**Keywords:** machine learning, channel quality classification, hardware implementation, FPGA

## Abstract

The latest generation of communication networks, such as SDVN (Software-defined vehicular network) and VANETs (Vehicular ad-hoc networks), should evaluate their communication channels to adapt their behavior. The quality of the communication in data networks depends on the behavior of the transmission channel selected to send the information. Transmission channels can be affected by diverse problems ranging from physical phenomena (e.g., weather, cosmic rays) to interference or faults inherent to data spectra. In particular, if the channel has a good transmission quality, we might maximize the bandwidth use. Otherwise, although fault-tolerant schemes degrade the transmission speed by solving errors or failures should be included, these schemes spend more energy and are slower due to requesting lost packets (recovery). In this sense, one of the open problems in communications is how to design and implement an efficient and low-power-consumption mechanism capable of sensing the quality of the channel and automatically making the adjustments to select the channel over which transmit. In this work, we present a trade-off analysis based on hardware implementation to identify if a channel has a low or high quality, implementing four machine learning algorithms: Decision Trees, Multi-Layer Perceptron, Logistic Regression, and Support Vector Machines. We obtained the best trade-off with an accuracy of 95.01% and efficiency of 9.83 Mbps/LUT (LookUp Table) with a hardware implementation of a Decision Tree algorithm with a depth of five.

## 1. Introduction

Today, the world is becoming interconnected, the number of devices is increasing exponentially, and the amount of data is overgrowing, being transmitted and received over wired and wireless infrastructure. These data contain valuable information that allows diverse fields to stay updated and operate correctly to fulfill various necessities.

**Figure 1:**
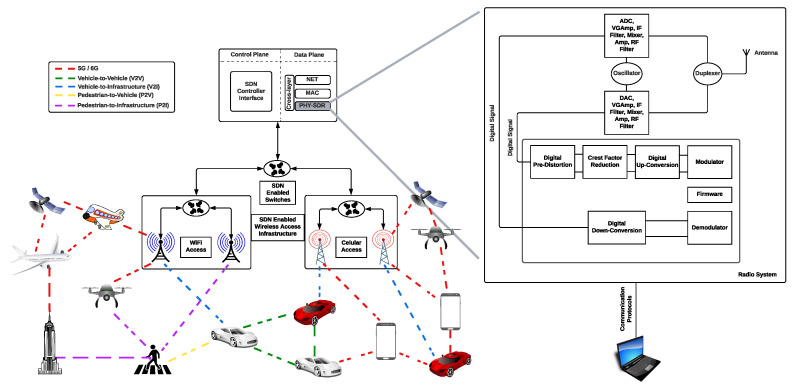
An operating environment of communication networks based on SDR.

Recent networks, such as 5G, 6G, VANET, SDN, and SDVN, are focused on combining heterogeneous networks to offer novel, flexible, and dynamically (re)configurable network elements. This allows for providing diverse and customizable services to dynamic traffic demands in frequency, space, and time while satisfying user Quality of Service (QoS) requirements. Specifically, SDN enables one to abstract the network logic from a hardware implementation into software, separating the data and control planes with a controller to handle various applications, coordinating the network’s operations, and deciding the policies such as configuring the traffic and having a global view of the network to satisfy the QoS. The controller logic is implemented in the server as a software component, while the data plane logic is executed in the networking devices such as switches and routers [1], see Figure 1. The data plane forwards the packets to the appropriate destination, and one of these networks can be seen as an architecture that controls the network devices and controls the entire network. In this way, we can implement an SDR (Sofware Defined Radio) to carry out the programmability in the data plane, and an important application is to enable more efficient spectrum utilization through opportunistic use of the spectrum. Current SDR technology may act as an enabler, allowing researchers to design and develop new communication network technologies with special requirements focused on a software definition and allowing a cognitive radio to configure the transmission parameters of a device dynamically according to the environment in which it operates [1,2].

In this sense, in Vehicular Ad hoc Networks, the open problem is how to evaluate channel quality to change the communication channel or modify its functions according to the conditions. If there is low quality, then they have to use an error-correction module or better modules in the radio hardware or other algorithms in the processor for fault tolerance. The architecture of this network must integrate different types of networks to provide a platform for communications within the infrastructure and vehicle–infrastructure communications. This architecture allows the creation of various kinds of networks such as V2V (vehicle-to-vehicle), V2I (vehicle-to-infrastructure), V2X (vehicle-to-all ), V2P (vehicle-to-pedestrian), V2H (vehicle-to-home), V2B (vehicle-to-buildings), V2C (vehicle-to-the cloud), and 5G/6G [3,4,5].

The operating environment or the integrated applications environment of these communication networks carries many challenges, such as intermittent connectivity, the provisioning of quality of service (QoS), and the heterogeneity of applications when a vehicle is in motion or immobile [6]. Nevertheless, we expect those types of communication networks to continue working, offering different multimedia services such as web navigation, streaming, social networks, and routine operations of the vehicle. SDR, with its various components, can help solve these problems, for example, the channel quality, although new architectures must be proposed and evaluated.

An important parameter is the Network Channel Quality Indicator (CQI) that is used to cope with channel variations [7]. CQI is modeled mainly with three different types of phenomena: slow fading, multi-path fading, and noise [8,9]. To achieve better communication, devices must evaluate the quality of the communication channel, enabling the possibility of having fault-tolerant schemes or offering information to change the channel. Furthermore, concerning the provisioning of service quality, the physical level of communications is exposed to cosmic rays, lightning, and temperature changes, among others, which might cause noise and interference in the quality of the channel.

In this way, vehicular communication networks search for driver safety and traffic efficiency on the road by interchanging traffic-related information among vehicles and infrastructures, considering security requirements and efficiency in performance for improving traffic management in the future [10]. Hence, CQI is a critical factor for data exchange. For example, intelligent transportation systems need to exchange data with their neighbors about the position, speed, velocity, acceleration, and brake status, among others [11]. Machine-to-Machine and Human-to-Machine connectivity requires identifying, locating, tracking, monitoring, and controlling [12,13]; the vehicle-to-home (V2H), vehicle-to-vehicle (V2V), and vehicle-to-grid (V2G) concepts have energy-efficient requirements of power grids [14]; additionally, cooperative systems for infrastructure-to-vehicle communication aim to ensure safe and efficient driving in the increasingly overloaded infrastructure [15]. In these environments related to ad hoc networks, every node inside the network that communicates with each other is responsible for dynamically detecting available nodes by making rapid changes in connectivity [16].

In general, the trend in communication networks is to generate, transmit, store, and process large amounts of data, which can come from users, vehicles, or infrastructure. Specifically, it is essential to integrate systems capable of provisioning service quality to react to this enormous amount of infrastructure data, making an intelligent use of the available resources and leading to the application of Machine Learning (ML), a branch of Artificial Intelligence (AI), to identify and solve communication problems reactively. Hence, SDR has architectures that can be modified through components for examining channel quality and exploring the most suitable model among a wide range of options applied to the current problem: The most accurate models usually are not very explainable (e.g., support vector machines, deep neural networks, boosted trees, and random forests), and the most interpretive models are typically less accurate (e.g., linear or logistic regression) [17]. In addition, for hardware development, ML algorithms are computationally expensive.

Several works have studied how to perform trade-offs for a wide variety of applications, such as error-resilient, Sobel edge detector, Finite Inverse Response filter, Discrete Cosine Transform, and with artificial intelligence algorithms [18,19,20], among others, always searching to find the best balance between different parameters, i.e., accuracy, and energy-efficiency. Therefore, implementations in platforms such as Graphics Processing Units (GPUs), Field-Programmable Gate Arrays (FPGAs), and Application-specific Integrated Circuits (ASICs) are enforced to support the application of AI and ML algorithms [21], facilitated thanks to the increase in computing power, the number of sensors, available data, transmission bandwidth, latency, and falling costs [22]. The FPGA offers an advantage over fixed application-specific integrated circuit implementations due to its re-programmability, allowing hardware designs to be upgraded or re-purposed after deployment. It also provides dynamic re-programmability, where the function changes at run time in response to the requirements of the application, and supports partial reconfiguration, where only parts of the hardware are modified at run time [23].

In this article, we present a trade-off analysis of four FPGA hardware designs for a co-processor in an SDR architecture, which enables us to classify low and high CQI based on ML models and the eurecom elasticmon 5G 2019 dataset [24]. The dataset uses 4G/5G statistics and monitoring data. The four FPGA architectures are implemented the ML models, namely, Decision Trees, Multi-layer Perceptron (MLP), Logistic Regression, and Support Vector Machines (SVM). The CQI classification makes it possible to sense the communication environments and react according to user’s necessities since hardware components are susceptible to noise and radiation, reflecting a low CQI. The design and development of the FPGA hardware architectures have several challenges focused on research for satisfying different requirements such as low energy consumption, high performance, small hardware resources, and high efficiency since the initial designs are hardly applied directly. We can summarize the main contributions of the paper as follows:

- A hardware architecture proposed to be part of the components of a radio system. Its output can help change functions or algorithms for the modules and processors of the system itself to detect high or low quality in the communication channel, allowing it to sense the environment and adapt to its conditions. To the best of our knowledge, no studies have investigated using the eurecom elasticmon 5G 2019 dataset in this manner;
- Design and development of FPGA hardware architectures to implement four ML algorithms that classify low and high CQI: (a) two Decision Trees with depths of five and six; (b) a three-layer MLP with a configuration of 5-5-1, using an ReLU activation function in the first two layers and a Sigmoid activation function in the third layer; (c) a Logistic Regression architecture which comprises three modules; and (d) an SVM architecture consisting of two modules;
- A trade-off analysis of the FPGA hardware implementations for a co-processor in an SDR architecture comparing models based on four different ML algorithms in terms of accuracy, objectives, platforms, latency, Hardware Resources, Frequency, Throughput, and Efficiency.

The paper is structured as follows: Section 2 presents several studies of Decision Trees, Neural Networks, Logistic Regression, and SVM. Section 3 gives an overview of the used ML Algorithms. Section 4 describes our Machine Learning architectures and the shared basic modules. Section 5 shows resources comparison of the developed ML architectures and with related works. Finally, we conclude in Section 6.

## 2. Related Work

In recent years, the advancements in Machine Learning have made many processes easier, automating critical technologies nowadays. Embedded applications have important characteristics such as latency or local analysis rather than sending raw data to the cloud in a wide variety of applications. ML algorithms implemented in hardware offer an incredible capacity for developing systems for current meet demands and increase the possibilities in the future. In this way, we reviewed the development of the four implemented machine learning algorithms on FPGA (Decision Tree, Artificial Neural Networks, Logistic Regression, and SVM) in different works to measure resources comparison analyzing the potential of their application.

Lin Z. et al. [25] developed a quantile-based algorithm that modifies the induction of the Hoeffding tree for building an online Decision Tree learning system on an FPGA. They used a 32-bit fixed-point data representation for tackling the problem of learning large-scale data sets. The authors evaluate the design over five different data sets: (1) Bank Marketing, (2) MAGIC Gamma Telescope, (3) Australian New South Wales Electricity Market, (4) Covertype, and (5) Person Activity. The authors conclude that their proposed algorithm outperforms the Hoeffding tree learning method, leading from a 0.05% to 12.3% improvement in inference accuracy.

Choudhury et al. [26] proposed a 32-bit serial architecture for implementing a Two Means Decision Tree on a Virtex Ultrascale+ FPGA that provides reconfigurability and dynamic training. The architecture was proven on five binary balanced data sets: (i) skin, (ii) occupancy, (iii) activity recognition 1, (iv) activity recognition 2, and (v) mammography. The authors mention that their implementation runs 28X faster and has lower complexity than the C4.5 algorithm and that, in the future, they will search to implement parallel and pipelined versions.

Novickis R. et al. [27] developed two Feed-Forward Neural Networks (FFNN) by adopting a pipelining design technique aiming to estimate vertical forces on the four wheels of the electric car, receiving as inputs the steering angle, longitudinal acceleration, vertical acceleration, roll rate, pitch rate, yaw rate, and lateral acceleration derivative from an automative simulator called Dynacar. The FFNNs are split into elementary layers characterized by their resource and latency, such as an adder, multiplier, and activation function. The authors reported a mean absolute error of 0.0232 and 0.0189 for the two models.

Kachris C. et al. [28] developed a hardware accelerator for Logistic Regression. It was incorporated into a framework for data analytics called Spark. The hardware accelerator was written in C using the Xilinx Vivado High-Level Synthesis tool. The authors stated that the proposed work could be implemented in high-performance systems to reduce energy consumption and execution time. It can achieve up to 40 times speedup in embedded systems compared to embedded processors.

Batista G. et al. [29] proposed an asynchronous non-linear pipeline architecture of SVM classifier for speech recognition of 30 different classes aiming at low-power applications using a Gaussian kernel function. The architecture consists of four stages with a Multiply-Accumulator unit application and three different control circuits. The classifier was applied to 60 speeches and 20 speakers, obtaining 98% accuracy. The authors mention that their proposal is a promising solution for pattern recognition systems in low-power circuit applications due to its accuracy in power consumption, recognition success rate, response time, and circuitry area occupancy.

Wu R. et al. [30] presented an accelerator architecture for matrix computing method with changeable dimensions using SVM; this was achieved following the next steps: (i) the matrix data are stored as an external memory; (ii) the problem of the limited resources due to the matrix dimension is solved by the data mover of programmable logic, which transmits data to the processing element with the control of processing system; (iii) the transmission adopts a synchronous data mechanism; (iv) the architecture of the processing element is optimized to improve the data transmission performance and the effective utilization ratio of the floating-point unit; (v) multiple processing elements are implemented on the programmable logic; and (vi) multiple matrices are processed in parallel.

Zhang Y. et al. [31] proposed a strategy based on an SDVN framework to schedule the start-sending time of each transmission. The aim was to maximize the packet delivery ratio (PDR) while meeting QoS requirements of transmission delay, avoiding issues of time-slot allocation and synchronization. For this purpose, they developed an algorithm called TSGS focused on finding the optimal sending time for each connection with higher PDR while meeting QoS requirements for transmission delay. Their results show that they could effectively improve the PDR by at least 15%, enhance the collision-avoidance performance by almost 40%, and reduce the MMP ratio by about 3% compared with the random transmission.

We have described several applications that use different ML algorithms above, demonstrating the wide variety of fields that need intelligent systems to fulfill specific necessities. However, to the best of our knowledge, the classification of low and high CQI for offering noise detection for SDR using the eurecom elasticmon 5G 2019 has not been approached.

## 3. Preliminaries

The term Artificial Intelligence (AI) is applied to develop systems endowed with intellectual functions characteristic of humans, such as reasoning, discovering meaning, generalizing, or learning from experience [32,33]. A branch of AI is ML, which focuses on studying computer algorithms that improve automatically through experience. The ML algorithms acquire new knowledge through the combination of different processes, such as acquiring significant concepts and understanding their meanings and relationships. By using ML algorithms, we can solve problems such as classification (help in the prediction of some class), regression (help in the prediction of some continuous variable), association (find relations among variables in data sets without labels), or clustering (create the clusters based on the characteristics of the data points) [34,35].

Machine Learning algorithms are widely used for classification, which seeks to learn the relationships between a set of features for predicting a class. The algorithm receives a set of inputs and produces a classification result. There are two possible results (classes) for a binary case, where it is generally searched to answer a yes/no question. For example: Does the patient have a malignant tumor? Is the received email spam? Will the customer receive a credit? Is the channel quality adequate to transmit data? Is the channel quality low? In this paper, we are interested in four classification algorithms such as Logistic Regression, Support Vector Machines, Decision Trees, and Artificial Neural Networks(ANN) to determine the low or high quality of the communication channel.

An important factor in the performance of ML algorithms is the quality of the data, so raw data pre-processing is a fundamental step. This process is focused on selecting essential characteristics, eliminating noisy values, and filling missing values [36]. Subsequently, the data are generally divided into two sets, training and testing, where the first one serves to train the model, modifying the parameters of the algorithm. In contrast, the algorithm uses the second set to check if it can correctly classify data that it has not seen.

We describe in the following the four algorithms used for classifying high or low signal quality to detect if there is noise in the environment, which exists if CQI is low.

Decision Trees are constructed by a direct graph G=(V,E), $E\subset V^{2}$ with a set *V* of finite nodes, which is split into three disjoint sets V=D∪C∪T, where set *D* (decision nodes) selects an action; set *C* (chance nodes) selects randomly one of the edges stemming from a reaction; and set *T* (terminal nodes) represents the end of a sequence of actions and reactions [37]. Nodes are represented by figures: decision nodes (*D*) by squares, chance nodes (*C*) by circles, and terminal nodes (*T*) by triangles. Artificial Neural Networks (ANN) are structures inspired by the principles of the brain functions. ANN searches for a way to interpret knowledge so that networks can learn, generalize, and abstract concepts and relations [38]. ANNs have high parallelism and extreme robustness and, in general, have obtained good results in areas of interest such as medicine [39] and economics [40], among others. An error function is defined to determine if the obtained output equals the desired output for the training parameters. The error function needs to be minimized through the delta rule shown in Equation (1):

(1) $$\begin{array}{c} \theta^{new}=\theta^{old}-\eta \left(o-y\right)\\ W^{new}=W^{old}+\eta \left(o-y\right)X \end{array}$$

where η is the training rate, *o* is the desired output, and *y* is the actual output; the parameter η must be chosen carefully since a huge value can give convergence errors in large steps; in addition, if a very small value is selected, many resources can be occupied. General Neural Networks are defined as a directed graph, which is a pair G=(V,E) consisting of a finite set *V* of nodes and a finite set E⊆V×V of edges. An edge e=(u,v)∈E is said to be directed from vertex *u* to vertex *v*. Let G=(V,E) be a directed graph, and u∈V be a vertex; the vertices of set pred(u)=v∈V|(v,u)∈E are called predecessors of vertex *u*, and vertices of set succ(u)=v∈V|(u,v)∈E are called successors of vertex *u*. An artificial neural network is a directed graph G=(U,C) whose vertices u∈U are called neurons or units and whose edges c∈C are called connections; set *U* of vertices is divided into set $U_{in}$ of input neurons, set $U_{out}$ of output neurons, and $U_{hidden}$ set of hidden neurons (Equation (2)):

(2) $$U=U_{in}\cup U_{out}\cup U_{hidden}$$

Two large groups of artificial neural networks are distinguished according to their structure, which can be understood as a graph: (i) the first one is when the graph is acyclic (no loops). This type of network is called a feed-forward network, when information can only be transmitted in one direction; (ii) the second is defined when the graph is cyclical, called a recurrent network. This configuration presents feedback and depends on input and output values from previous steps. The computation of the neural networks is divided into input and work phases: the input phase consists of reading input values of the network and initializing weights in a random range with a small value. In contrast, the work phase computes the output.

Logistic Regression is a statistical model that uses a logistic function to model a dichotomous variable (binary). Logistic Regression determines a boundary between classes and determines class probabilities, depending on the distance from the edge [41]. The input values $X_{i}$, where *i* = 1, 2, …, *m*, are combined linearly using weights $\beta_{j}$ (also called coefficients), where *j* = 0, 1, 2, …, *m*, (see Equation (3)) to predict output *Y* (see Equation (3)):

(3) $$\begin{array}{c} X=\beta_{0}+\beta_{1}X_{1}+\beta_{2}X_{2}+\ldots +\beta_{m}X_{m}\;\\ Y\left(X\right)=\frac{1}{1+e^{-X}} \end{array}$$

Time complexity of Logistic Regression during training is calculated using *n* number of training examples and *d* number of dimensions, such that O(n·d), whereas space complexity is calculated using *d* number of dimensions (O(d)).

Support Vector Machine belongs to the supervised learning algorithms that can be used for classification and regression problems. It is based on the idea of mapping data points from low dimensions to high dimensions using kernel functions [42], such as Linear, Polynomial, RBF, and Sigmoid, shown in Equation (4):

(4) $$\begin{array}{c} f\left(x\right)=x_{i}^{t}x_{j}\;\\ f\left(x\right)={\left(\gamma x_{i}^{t}x_{j}+r\right)}^{t}\;\\ f\left(x\right)=exp(-\gamma ||x-x^{\prime }|{|}^{2})\;\\ f\left(x\right)=tanh\left(\gamma x_{i}^{t}x_{j}+r\right)\; \end{array}$$

The coefficient γ is a conditional hyperparameter when the kernel is set to polynomial, RBF, or sigmoid; *r* is a conditional hyperparameter of polynomial and sigmoid kernels; *d* is a hyperparameter that represents the degree of the polynomial kernel function. The complexity of SVM during the training phase scales between $O(n_{features}\cdot n_{samples}^{2})$ and $O(n_{features}\cdot n_{samples}^{3})$.

## 4. Methods

**Figure 2:**
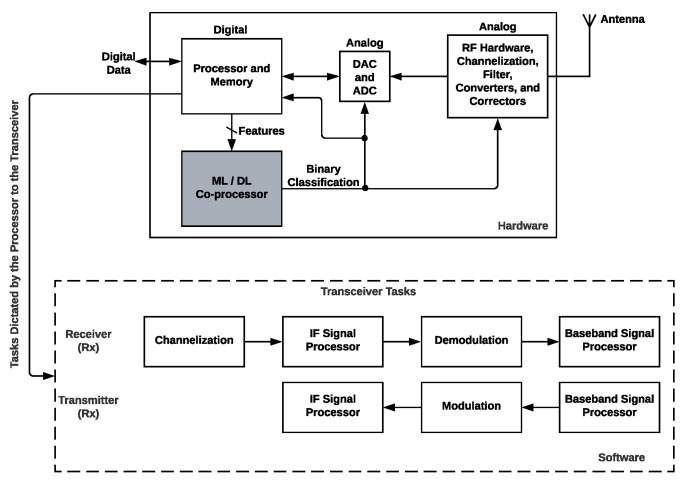
Proposed radio system based on SDR and the ML/DL Co-processor.

The proposed architecture is a radio-system component that we have considered as an ML(Machine learning)/DL(Deep Learning) co-processor that allows its use in an SDR scheme, see Figure 2. The processor receives the flow of the data packets; evaluates the conditions; and measures, extracts, and sends the features to the ML/DL co-processor. Four hardware architectures to build the co-processor based on an FPGA in this article are examined, designed, implemented, and compared with other works based on hardware and software implementations. Our co-processor allows us to evaluate the features sent by the processor to determine if the channel quality is low or high. The components can use this output to modify their hardware error correction modules, filters, operating channel processes, converters to change their precision and accuracy, improving QoS. In addition, the digital hardware component can select better fault tolerance algorithms (software), modulation schemes, and transforms that adapt best among other algorithms or functions. By considering these conditions, we focus on having an SDR that can modify its hardware and software in most aspects. The advantages of the proposed architecture in Figure 2 are changing between algorithms that can consume less energy to others that consume more energy but provide:

- Tolerant communication schemes;
- Capability to notify in a context of the change of channel;
- Provide better conditions of communication bandwidth or less noise.

In the following sections, we describe the co-processor development. We will explain how we selected the machine learning model that determines if a channel has a high or low quality based on a trade-off analysis. The learning-model implementation is based on an FPGA hardware architecture, which is part of an SDR.

### 4.1. System Model

SDR offers many advantages that networks such as SDVN, SDN, V2V, and V2I can exploit since SDR is a complete system, and each base station accesses the radio functions, where hardware devices with reconfiguration are important elements. These technologies must enable solving disadvantages that SDR cannot solve, such as scheduling problems, resource allocation, troubleshooting, load balancing, and security. In this way, our work is focused on the fact that if we have a communication network technology such as SDVN based on SDR, then we can evaluate and measure different characteristics to determine the quality of the channel so that this type of system can adjust security, resource management, improvements in the use of bandwidth, and power consumption, among others.

**Figure 3:**
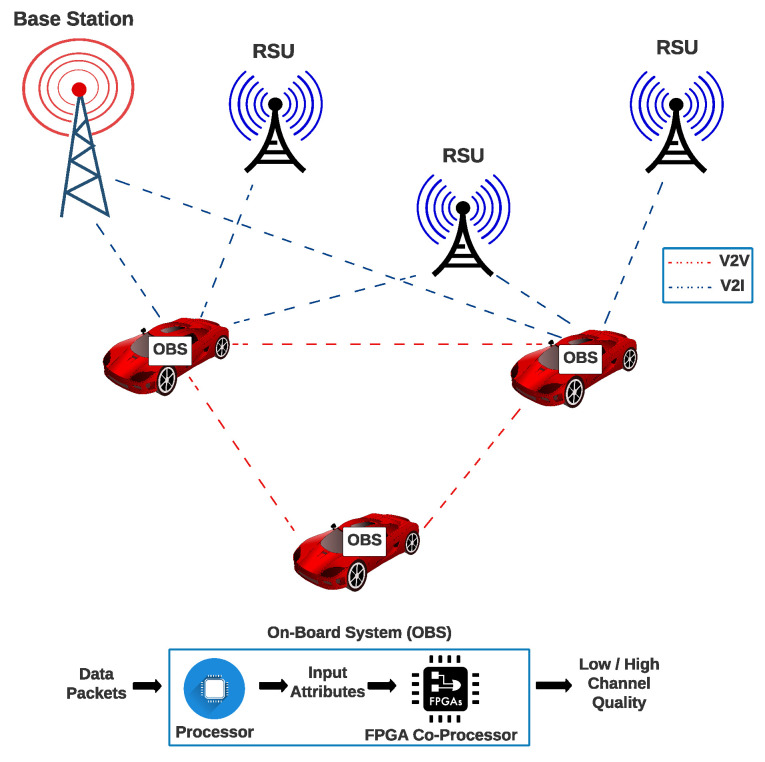
System model to implement in V2V and V2I Communications our proposal as part of an On-Board System.

This work used the database Eurecom/elasticmon5G2019 for SDR to evaluate the channel quality. Our proposal can be implemented as an OBS (On-Board system) integrated with the vehicles that operate in networks such as SDVN or VANET, see Figure 3. We remark that the devices that integrate an SDVN or VANET included in the system model are affected by noise and interferences that decrease the Quality of Service of the channel transmission. In addition, our proposal is evaluated in FPGA. Still, it is important to mention that there are Commercial Reconfigurable Processors [43] or Systems on Chip integrating FPGA [44,45,46,47] and FPGAs that have been integrating Processing Cores for years. Therefore, it is possible to integrate our solution in vehicular systems as well as in industrial, business, or home systems, as we show in Figure 3.

We implemented four hardware architectures based on Decision Trees, MLP, Logistic Regression, and SVM for CQI classification. We selected these algorithms due to the following advantages: Decision trees do not require feature scaling (standardization and normalization) since they are based on rules instead of distance calculation. Neural networks are robust against noise since they are formed by individual units called neurons working in parallel, allowing correct functioning even if some unit fails. In addition, Neural networks capture optimal and effective complex characteristics, obtaining results with high precision. Logistic regression is easy to implement, interpret, very efficient to train, and is less inclined to over-fitting. SVM is effective in high dimensional spaces and uses a subset of training points, being memory efficient.

Therefore, it is crucial to identify the common operations involved in the four machine learning algorithms and implement them in modules to reutilize each one. This section first introduced the standard components and how they are implemented. We describe the decision trees’ architecture based entirely on comparators; analogously, the decision tree architecture is also based on an algorithm formed of if-else nested instructions. Multipliers, adders, comparators, and the modules of activation functions were used for the different layers form the MLP architecture, which is the most complex implementation. Finally, Logistic Regression and SVM are based on the re-utilization of the modules described. The data are represented using the standard IEEE 754 for Floating-Point Representation of 32 bits [47].

### 4.2. Common Components

The common hardware modules used in MLP and Logistic Regression architectures are multipliers, adders, comparators, and the Sigmoid module. Multipliers and adders are the most used components. We developed a single module containing the above operations, avoiding making several copies of multipliers and adders for MLP, Logistic Regression, and SVM.

**Figure 4:**
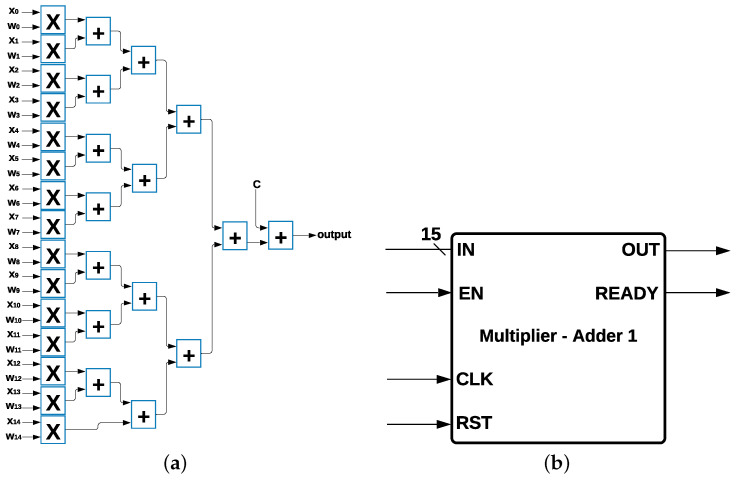
Multiplier Adder Module 1 and its Components. (**a**) Common Multipliers and Adders. (**b**) Multiplier Adder Module 1.

Multiplier Adder Module: MLP, Logistic Regression, and SVM have 15 multipliers and 15 adders as common components (Figure 4a) in their architecture, and each multiplier has two data inputs: $x_{i}$ and $w_{i}$ with i=0,1,2,…,14, where $x_{i}$ are the 15 features from the data set, and $w_{i}$ is the weight multiplied by the inputs, such that $x_{i}\times w_{i}$ for every *i*; in addition, there is a value *c* that is calculated depending on the algorithm. The output was determined by using the Equation (5):

(5) $$output=(\sum_{i=0}^{14}x_{i}w_{i})+c$$

The 15 multipliers and 15 adders were represented as a single module with 15 data inputs and 1 data output and 3 control signals called *enable* (EN), *reset* (RST), and *ready*, which indicate when the process has been completed and the output is ready to be communicated. This module is called Multiplier-Adder Module 1, shown in Figure 4b.

Sigmoid Module: The *Sigmoid* activation function is part of MLP and Logistic Regression architectures. Different steps are necessary for the hardware implementation of the *Sigmoid* function since it is not possible to implement it directly. *Sigmoid* has a range of between zero and one, and its expression is shown in Equation (6):

(6) $$f\left(X\right)=\frac{1}{1+e^{-X}}$$

**Figure 5:**
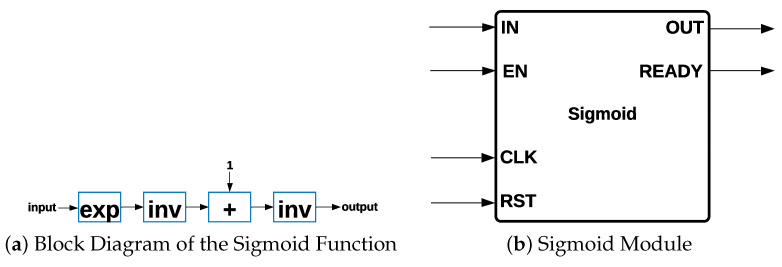
Block Diagram and Module of the Sigmoid Function.

The block diagram of the *Sigmoid* activation function is shown in Figure 5a, such that the process consists of four different steps: (i) $e^{x}$ is calculated, such that the input value is the exponent of the Euler constant *e*; (ii) the result of $e^{x}$ is inverted, obtaining a result of $1/e^{x}=e^{-x}$ to this point; (iii) a value of one is added to the previous result, forming the denominator of the *Sigmoid* activation function; and (iv) the result of $1+e^{-x}$ is inverted. The representation of the Sigmoid module is shown in Figure 5b.

**Figure 6:**
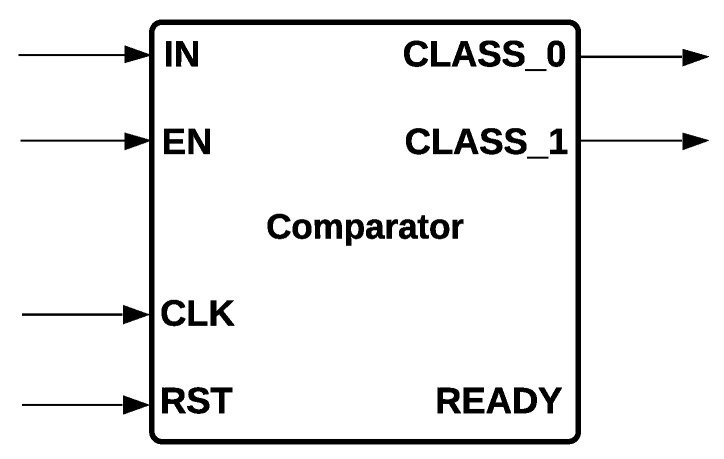
Comparator Module of MLP and Logistic Regression.

Comparator Module: It is the common component of MLP and Logistic Regression that determines the class to be activated: class zero or class one. The module has one data input; one store value; three control signals, EN, RST, and READY; and two outputs, *CLASS_0* and *CLASS_1*, that are determined depending on the input signals. *CLASS_0* is activated if the input is bigger than 0.5; if not, *CLASS_1* is activated. The comparator module is shown in Figure 6. We remark that a comparator is also used for Decision Trees and SVM. However, their implementation is slightly different; for example, in Decision Tree, there are two outputs that activate the left or right node, respectively, whereas SVM implements two comparators for forming a Hard Margin.

### 4.3. Dataset Description

Eurecom/elasticmon5G2019 is the dataset used for training and evaluating the machine learning models that carry out a binary classification. The dataset contains the following attributes: 4G/5G MAC (Media Access Control), RRC (Radio Resource Control), PDCP (Packet Data Convergence Protocol) statistics. The dataset was constructed by monitoring 100 features per measurement, storing a total of 26082 examples. A pre-processing stage was executed, and 5 steps were followed: (1) a timestamp for the measurements was implemented, (2) stable features were deleted from the dataset, (3) overflows and metric spikes were removed using the mean or median of the other values, (4) a correlation matrix was applied in order to know the features that correlate the most with CQI, (v) the top 15 features were selected (see Table 1a), and (vi) the dataset was scaled between 0 and 1, which is required for training some models due to convergence being more difficult if data are not scaled.

The dataset was divided into 70% for training and 30% for testing; however, a third set called *validation* is contained inside the *training* set since we used k-fold cross-validation with k=10. The ML algorithm is trained with 9 out of 10 parts of the *training*, and the accuracy is measured over the remaining one-tenth of the set, which is the *validation*. This process is iterated 10 times, and a global accuracy for the *training* is obtained by averaging the accuracy over the 10 *validation* sets. The labels to classify the channel quality were assigned according to the technical specification of the 3rd Generation Partnership Project (3GPP) [48], where a CQI from 0 to 6 is considered low quality and a CQI from 7 to 15 is considered high quality, see Table 1b.

### 4.4. Hyperparameter Configurations

One of the more common techniques for examining an algorithm hyperparameter configuration is to establish the exploration sets manually [49]. Continuous and discrete hyperparameters allow controlling the training process by regulating the behavior of the ML algorithms. We implemented several experiments considering a range of hyperparameters (manually established), Table 2. We selected the best configuration for each ML algorithm with the balanced accuracy metric as a target, implementing a grid search to carry out the hyperparameter tuning task to obtain the highest score using cross-validation folds. The final configuration for each ML algorithm is shown in column Configuration of Table 2. Python was the selected programming language since it contains specialized libraries for Machine Learning. Python was executed on a cloud service called Google Colab, allowing the use of CPUs and GPUs from Google. In this case, the provided equipment was an Intel Xeon CPU 2.20GHz.

For finding the hyperparameters described in Table 2, we followed the subsequent process: (1) The ML algorithm was selected: Random Forest, Decision Tree, MLP, CNN, Logistic Regression, or SVM; (2) the hyperparameter range for the selected ML algorithm was established, and we established different values for every hyperparameter to explore several combinations; (3) techniques for imbalanced datasets were established, and we applied two types, data-level (random undersampling, random oversampling, and SMOTE-Tomek [50]) and algorithm-level (balanced weights and cost-sensitive); and (4) for each technique toward imbalanced data sets, a hyperparameter search was implemented using the selected ML algorithm, its hyperparameter range, stratified k-fold cross-validation, and the balanced accuracy for evaluation. The ML algorithm was trained with the first hyperparameter combination using stratified k-fold cross-validation with k=10 over the training set. The process was repeated until all possible hyperparameter combinations had been evaluated.

### 4.5. Decision Trees

**Figure 7:**
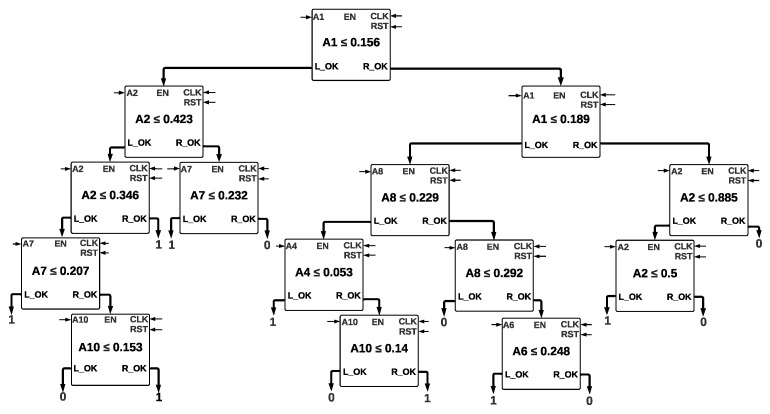
Decision Tree with a Depth of Five.

For the Decision Tree, the used technique was balanced weights, with the next hyperparameters: the entropy criterion, a maximum depth of 6, a minimum samples split of 2, a minimum samples leaf of 3, and a minimum weight fraction leaf of zero. Every node is formed by a comparator, which has 2 inputs of 32 bits each: *A* and *B*. The outputs are called *L_OK* and *R_OK*, which activate left and right nodes, respectively. If condition A≤B is fulfilled, *L_OK* takes a value of one, and the left node is activated; if the condition is not fulfilled, *R_OK* takes a value of one, and the right node is activated. This condition is repeated for every node until a leaf is reached and the class is determined. The tree architecture with a depth of five is shown in Figure 7, where it is shown every value that is stored inside every comparator.

The inputs $A_{1}$, $A_{2}$, $A_{4}$, $A_{6}$, $A_{7}$, $A_{8}$, and $A_{10}$ correspond to the features of the dataset. We implemented two Decision Trees with depths of six and five. The Decision Tree’s complexity with a depth of six is bigger since it contains more nodes to evaluate. We pruned both trees, considering that if two leaves of a certain node belong to the same class, they are eliminated, and the node turns into a leaf with the class value.

### 4.6. Multi-Layer Perceptron

**Figure 8:**
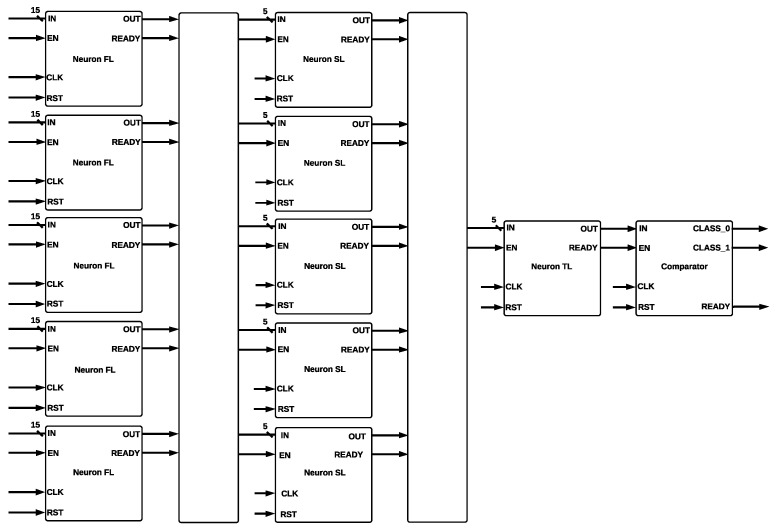
Neural Network Hardware Architecture.

The MLP comprises three dense layers using the SMOTE-tomek technique [50] with the following hyperparameters: five neurons for the first two layers and one neuron at the output layer since the problem is binary. An ReLU was the activation function for the first two layers, and a sigmoid function was used at the output layer. The RMSprop optimizer, with a batch size of five, demonstrated that the algorithm achieved good results with a small number of samples before updating the parameters: a learning rate of 0.01, a glorot normal weight initialization, and a zero dropout. The MLP architecture contains three layers, input, hidden, and output layers, and is formed mainly by adders and multipliers. The architecture of the MLP architecture is shown in Figure 8. Adder and multiplier modules have two data inputs added or multiplied to obtain their corresponding output. The ReLU module was used at the first and second layers of the MLP design. Every neuron is represented as a single module with 15 inputs and 1 output; five neurons in parallel form the first layer. Every neuron of the second layer is represented as a single module with five inputs and one output; five neurons in parallel formed the second layer. A sigmoid activation function was used at the third layer, which contains one neuron, represented as a single module with five inputs and two outputs, called *CLASS0* and *CLASS1*, which indicate high or low quality, respectively.

**Figure 9:**
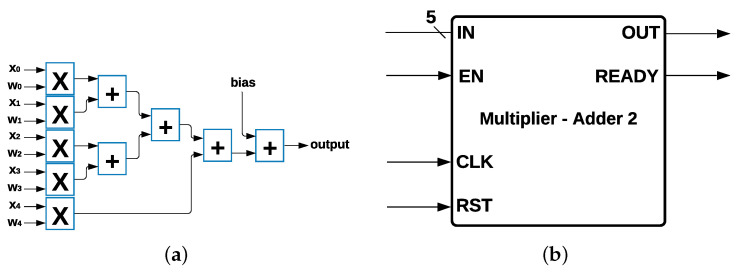
Multiplier Adder Module 1 and its Components: (**a**) Components of the Neurons of the Second and Third Layer; (**b**) Multiplier-Adder 2.

Every neuron of the second and third layers contains the same components except the activation function. In total, there are five multiplicative modules, represented by an *X*, and five addition modules, represented by “+”, as shown in Figure 9a. The five multipliers and the five adders are represented by a single module called *Multiplier - Adder 2* (Figure 9b), which has five inputs (*IN*); three control signals, (EN), (CLK), and READY; a CLK input; and one output (OUT).

**Figure 10:**
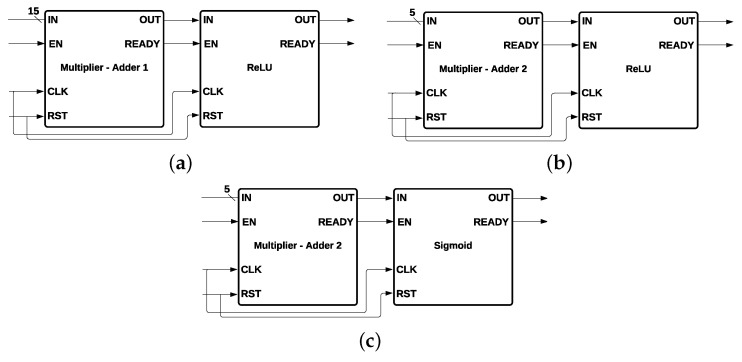
Neurons of the Feed-Forward Neural Network. (**a**) Neuron of the First Layer, 15-bit input and *ReLU* activation function; (**b**) Neuron of the Second Layer, 5-bit input and *ReLU* activation function; (**c**) Neuron of the Third Layer, 5-bit input and *Sigmoid* activation function.

The *ReLU* function was used for the second layer, and the *Sigmoid* function was used for the third layer. The output of the *ReLU* function is the maximum value between zero and the input value *X*; the output is zero when *X* is negative, and the output is equal to *X* when *X* is positive, such that relu(X)=max(0,X). Every neuron of the first layer (Figure 10a) is composed by a Multiplier-Adder 1 and a ReLU activation function; every neuron of the second layer (Figure 10b) is formed by a Multiplier-Adder 2 and an ReLU activation function; and the neuron of the third layer (Figure 10c) contains a Multiplier-Adder 2 and a *Sigmoid* activation function.

The *ReLU* activation function was formed by a comparator, which has a stored value of zero. Then, the input is compared, and if it is greater than zero, the output will take the value of the input; if not, the output will take a value of zero. Five neurons formed the first layer (input layer), which receives 15 inputs that correspond to channel quality information. The outputs of the first layer are connected to the inputs of the second layer (hidden layer), which also has five neurons. The outputs of the second layer are connected to the inputs of the third layer (output layer). The last layer determines which class will be activated, CLASS0 (high CQI) or CLASS1 (low CQI).

### 4.7. Logistic Regression

**Figure 11:**
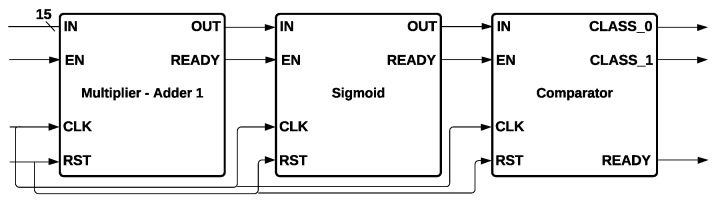
Logistic Regression Hardware Architecture.

In Logistic Regression, the used technique was SMOTE-tomek [50] and the following hyperparameters: an l2 penalty, a tolerance for stopping criteria (tol) of 0.0001, an inverse of regularization strength C of 10,000, the algorithm *newton_cg* for the optimization problem, and a maximum iteration of 1000, which is the number of iterations taken for the solvers to converge. Logistic Regression hardware architecture is formed by a Multiplier-Adder 1, a Sigmoid function, and a comparator, as is shown in Figure 11.

Multiplier-Adder 1 receives 15 inputs that give information about CQI. They were multiplied by the weights and added until a single value was obtained, which is the input to the *Sigmoid* module. Finally, the output of Sigmoid output was connected to the comparator input, which determines if CLASS0 or CLASS1 is activated, depending on if the output of *Sigmoid* is higher or lower than 0.5, respectively.

### 4.8. Support Vector Machine

For SVM, the contemplated technique was SMOTE-tomek [50], using as hyperparameters RBF (Radial Basis Function), a value of *C* = 464.1588, and a scale gamma value where the kernel function RBF uses these last 2 values. The parameter *C* trades off misclassification of training examples against the simplicity of the decision surface, whereas gamma defines how much influence a single training can have.

**Figure 12:**
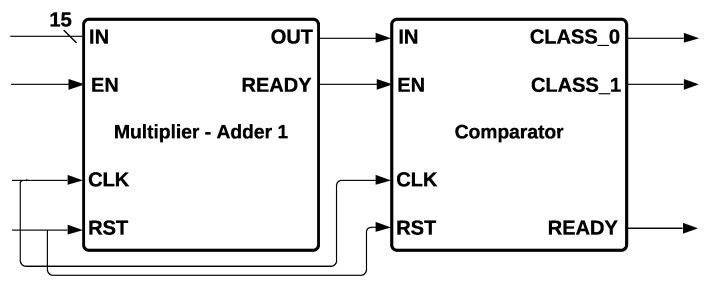
Support Vector Machine Hardware Architecture.

SVM Hardware Architecture is shown in Figure 12. The architecture is formed by a Multiplier-Adder 1 and comparators that act like Hard Margins. The 15 inputs were entered into Multiplier-Adder 1. Then, they were multiplied by the weights and added until a single value was obtained, compared with the Hard Margin, which determines if CLASS0 or CLASS1 is activated.

A hyperplane can be written as $(\sum_{i=0}^{k-1}x_{i}w_{i})-b=0$ for *k* dimensions. For Hard Margin, two hyper-planes were selected for separating two classes of data for this particular problem (high and low CQI classification), such that the distance between these two hyperplanes was calculated by 2/|w|, where |w| is the norm of *w*. The output was determined by Equation (7):

(7) $$output=\left\{\begin{array}{c} CLASS\_0\;\mathrm{if}\;(\sum_{i=0}^{14}x_{i}w_{i})-b\geq 1\\ CLASS\_1\;\mathrm{if}\;(\sum_{i=0}^{14}x_{i}w_{i})-b\leq -1 \end{array}\right.$$

The module called *Comparators* is formed by two comparators in parallel, such that it is determined if the output of the Multiplier-Adder 1 is equal or greater than one, or if it is equal or less than minus one.

## 5. Results

We divide the presented results into three subsections. The first two compare our four ML hardware implementations to measure the resources employed for each algorithm in terms of the following metrics: (1) latency: number of clock cycles counted from the beginning of algorithm execution until obtaining an output (low or high channel quality); (2) Look-Up Tables (LUTs): hold a custom truth table that is loaded when the chip is powered up; (3) Flip-Flops (FFs): basic memories for sequential logic operations; (4) Digital Signal Processor (DSP): a set of instructions for applications that require numerical operations at very high speeds; (5) Minimum Period: measured in *ns*, calculates the worst case path timing from clock edge to clock edge for flip-flops within the design; (6) Maximum Frequency: measured in MHz, it is obtained by calculating the inverse of the minimum period; (7) Throughput: measured in bps, it is the number of bits that can be processed per second; (8) efficiency: measured in bps/LUT, it is the ratio between the throughput and the number of LUTs; (9) Balanced Accuracy: in the binary case, it is described by the mean of sensitivity and specificity or by the area under the ROC curve with binary predictions rather than scores; (10) Sensitivity (also called recall): is the true positive rate and defines how well the positive class is predicted; and (11) Specificity: is the true negative rate and defines how well the negative class is predicted. In the third one, we compare our hardware implementations with previous works in terms of the resources mentioned before. However, the comparisons are not 100% fair since the implementations aim for different objectives using different datasets with variable sizes. As far as we know, there are no hardware implementations for the channel-quality classification model.

### 5.1. Resources Comparison

The number of resources utilized by the four developed ML algorithms is summarized in Table 3. It is essential to mention that the number of features could impact the number of resources employed. For example, for the implemented MLP model, there are 15 multiplier and 15 adder modules per neuron due to the 15 features. If more or fewer features are required, the number of modules will increase or decrease in proportion.

In terms of resources, the best results were achieved by the Decision Tree Algorithm with a depth of five, since it has the highest efficiency (9.83 Mbps/LUT), throughput (3148.76 Mbps), and frequency (323.31 MHz) and the lowest number of LUTs (320), FFs (812), DSPs (0), and period (3.09 ns). Its latency is 23 and was overcome only by two clock cycles (21) by Logistic Regression; however, the difference is small. The worst results belong to MLP since it has the lowest throughput (249 Mbps) and efficiency (0.006 Mbps/LUT) and the highest latency (317), LUTs (36825), FFs (79465), and DSPs (439).

For the comparison of metrics, in balanced accuracy, the highest score was achieved by the Decision Tree with a depth of 6, with 95.26, followed closely by the Decision Tree with a depth of 5 with 95.01. The lowest result was achieved by SVM with 92.06. However, the difference between the highest and lowest result is slight, with only 3.2. For sensitivity, the highest score (97.48) was obtained by MLP. The Decision Tree with a depth of 6 obtained 94.88, whereas the lowest belongs to SVM with 92.99. The difference of values between MLP and SVM is 4.49. For specificity, the highest score belongs to the Decision Tree with a depth of five with 96.91, followed closely by the Decision Tree with a depth of six with 95.64; the lowest result belongs to MLP with 90.57; the difference between the highest and lowest result is 6.34. We noticed that MLP has the highest imbalance for recognizing classes, since it has the highest sensitivity (true positive rate) but the lowest specificity (true negative rate). Decision Tree and CNN can recognize the negative class better than the positive class, whereas SVM recognizes the positive class better than the negative class. We remark that sensitivity and specificity are used for calculating balanced accuracy; for this reason, only balanced accuracy is used for trade-off comparison of the following sub-section.

### 5.2. Trade-Off Comparison

**Figure 13:**
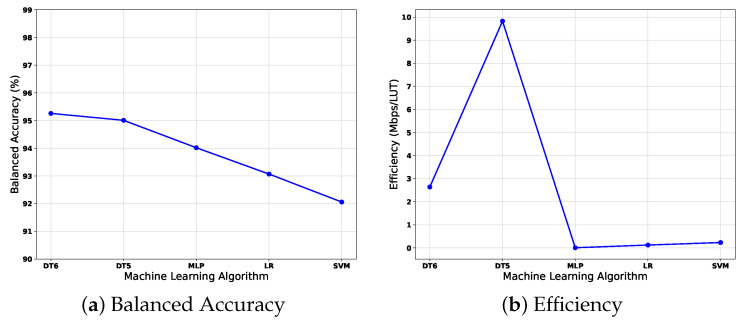
Trade-Off between Accuracy and Efficiency.

The trade-off comparison among developed ML algorithms is depicted in Figure 13a,b, where balanced accuracy and efficiency are shown. In this way, the goal is to determine which algorithm has the best compromise, which means a high balanced accuracy with high efficiency (Mbps/LUT).

The difference between the highest balanced accuracy (achieved by the Decision Tree with a depth of 6 and 95.26%) and the lowest (achieved by the SVM 92.06% is 3.19%) demonstrates that the developed algorithms had high performance. However, the efficiency difference between the highest (DT5 with 9.83) and the lowest (MLP with 0.0067) is 9.82; we consider this difference due to each algorithm’s type of implementation. On the one hand, Decision Trees architectures are based on comparators, avoiding adders and multipliers, which are expensive in terms of hardware. On the other hand, MLP, Logistic Regression, and SVM involve adders and multipliers. MLP especially requires 15 multipliers and 15 adders for every neuron, for a total of 165 multipliers and 165 adders, in addition to the activation functions (ReLU and Sigmoid). An ReLU is the activation function for the first two layers and a sigmoid function at the output layer. For these reasons, the efficiency is low for these three algorithms. Even if the Decision Tree with a depth of six has the highest balanced accuracy, the difference with the Decision Tree with a depth of five is minimal (only 0.2446%). If both efficiencies are compared, the Decision Tree with a depth of five is higher. Therefore, the Decision Tree with a depth of five has the best trade-off between balanced accuracy and efficiency.

### 5.3. Comparison with Other Works

As far as we know, there are no hardware implementations for the channel-quality classification model. Nevertheless, we compare our hardware implementations with previous works that have proposed machine learning algorithms for hardware implementations similar to our proposals in terms of the resources mentioned before. We remark that the comparisons are not 100% fair since the implementations aim for different objectives using different datasets with variable sizes, but we contrast the results to highlight our results achieved.

The four FPGA architectures that implemented the ML algorithms in this work (Decision Trees, NNs, Logistic Regression, and SVM) are compared with other implementations, see Table 4. Our work seeks to classify low and high CQI. In contrast, Lin Z. et al. [25] developed a Decision Tree aiming at large datasets, and Choudhury et al. [26] presented a Decision Tree with dynamic training, applied to five binary datasets. Novickis R. et al. [27] developed an FFNN for estimating vertical forces on the four wheels of the electric car. Kachris C. et al. [28] implemented a Logistic Regression architecture, which was incorporated into a framework for data analytics called Spark. Batista G. et al. [29] proposed an SVM classifier for speech recognition. Wu R. et al. [30] presented an SVM architecture for matrix computing with changeable dimensions.

The present work uses balanced accuracy for evaluating the results; Lin Z. et al. [25] reported five different results since they evaluated the design using the accuracy metric over five data sets: (i) Bank Marketing, (ii) MAGIC Gamma Telescope, (iii) Australian New South Wales Electricity Market, (iv) Covertype, and (v) Person Activity. Choudhury et al. [26] proved their architecture on five binary balanced datasets: (i) skin, (ii) occupancy, (iii) activity recognition, (iv) activity recognition, and (v) mammography. Their accuracy results correspond to these datasets. Novickis R. et al. [27] used the Mean Absolute Error metric to evaluate three different FFNNs configurations; Kachris C. et al. [28] and Batista G. et al. [29] evaluated their works using the accuracy metric; Wu R. et al. [30] chose the effective utilization rate for evaluation.

Two decision trees with depths of five and six were implemented in our work, whereas Lin Z. et al. [25] developed one architecture, but they implemented it on five different datasets. Choudhury R. et al. [26] developed one architecture, which was applied to five different datasets, obtaining the highest number of LUTs and FFs in this last work with 894,000 and 6488, respectively, followed by Lin Z. et al. [25] with a minimum number of LUTs of 54,198; however, they did not report the number of FFs. Our work obtained the minimum number of LUTs and FFs, where the Decision Tree with a depth of 5 has 320 and 812, respectively, and the Decision Tree with a depth of 6 has 890 and 1389, respectively. In the case of BRAMs, the maximum number was obtained by Lin Z. et al. [25] with 384, followed by Choudhury R. et al. [26] with 228, which also has the highest number of DSPs with 200, followed by Lin Z. et al. [25] with 138. In contrast, our work did not make use of any BRAM or DSP. The reported latency in our work is given in clock cycles (23 and 25) and milliseconds (ms), in contrast, Lin Z. et al. [25] with 0.26 and Choudhury R. et al. [26] with 24. The throughput and efficiency of our work achieve the highest frequency (MHz) with 323.31, followed by Lin Z. et al. [25] with 308 and by Choudhury R. et al. [26] with 62.

For Neural Networks, we presented an MLP model. In contrast, Novickis R. et al. [27] developed three FFNN models varying data type, activation function, and the number of iterations and obtaining three different results. We obtained a higher number of LUTS and DSPs, with 36,825 and 439, respectively, in contrast to Novickis R. et al. [27], which obtained 8881 LUTs and 14 DSPs, considering their best results. We reported the used memory in terms of FFs (79,465), Novickis R. et al. [27] in registers (22,123, best result). We did not use BRAMs, whereas Novickis R. et al. [27] used eight. We achieved a higher result with 164.44 MHz for frequency since Novickis R. et al. [27] achieved 100 MHz. We described the throughput in Megabits per second (Mbps) with 249, and Novickis R. et al. [27] in Mega samples per second (MSam/s) with 2.02 (best result). The achieved latency for this work is described in clock cycles, reaching 317, while Novickis R. et al. [27] used microseconds (8.05, best result).

For Logistic Regression, the achieved latency in our work is 21 clock cycles, whereas Kachris C. et al. [28] mentioned that it depends on the number of iterations that are performed by their system. In addition, their number of resources in terms of LUTs, FFs, BRAMs, and DPSs is higher than our proposal. The frequency of operation is 31.81 MHz for our work, and it is 667 MHz for the implementation of Kachris C. et al. [28], where the throughput and efficiency were not reported.

In the case of our SVM architecture, we implement a linear kernel, as well as the work developed by Wu R. et al. [30]. Our work achieved a latency of 25 clock cycles; Batista G. et al. [29] and Wu R. et al. [30] reported the latency in seconds, obtaining 1.648 us in the first and a range of 274.50–40632 ns (depending on the number of dimensions) in the second. The minimum number of LUTs was obtained by Batista G. et al. [29] (1314), followed by our proposal (4865) and Wu R. et al. [30] (12,090). The FFs and DSPs were not reported by Batista G. et al. [29] and Wu R. et al. [30], whereas the present work obtained 1922 FFs and 75 DSPs. For BRAMs, Batista G. et al. [29] did not report the results; in contrast, our architecture achieved zero BRAMs, followed by Wu R. et al. [30] (8). The maximum frequency was achieved by Wu R. et al. [30] (100 Mhz), followed by our proposal with 58.84 MHz and Batista G. et al. [29] with 50 MHz. We reported the throughput in Mb/s, obtaining 1129.8, whereas Batista G. et al. [29] reported it in MOPS, obtaining 1.64. Wu R. et al. [30] did not report it.

## 6. Conclusions

Next-generation communication networks require intelligent elements to adapt to their environment by evaluating their quality of service (QoS) and making decisions. One of these QoS factors is the signal quality of the channel through which the data are transmitted, known as the Network Channel Quality Indicator (CQI). In this case, the signal quality can be affected by multiple factors, such as the presence of cosmic rays, lightning, changes in temperature, and/or interferences. We can observe these effects, for instance, within vehicle applications where many devices and several communication networks coexist in the same environment. The signal quality is an important metric that helps to determine when the phenomena mentioned above affect services and generate interferences, causing erroneous behavior, failures, and more significant economic losses and even deaths. Real-time operating systems require continuous operation with great precision on the information exchanged.

Therefore, this article presented a trade-off analysis of four FPGA hardware architectures that implement the machine learning models of decision trees, MLP, logistic regression, and SVM to determine a high or low CQI. We carry out an analysis in software to obtain the machine learning models implemented. The FPGA hardware architectures trade-off analysis is focused on comparing performance metrics, resources used, and the learning model’s accuracy to assess the quality of the channel, which is applied in SDR in this work. So, future implementation in embedded systems will allow its application in larger integral systems within the entire range of communication networks, such as SDVN, SDN, 5G, and VANETs based on SDR. We have used mainly adder, multiplier, and comparator modules for the hardware implementation. We obtained quantitative values of the FPGA hardware implementations such as latency, LUTs, DSP, BRAM, throughput, and efficiency, together with the balanced accuracy metric, help us to select the best model. In this way, the results show that we obtained the best trade-off between balanced precision (ML model result) and performance (hardware result) with the decision tree, which consists of a depth of 5, a precision of 95.01%, and an efficiency of 9.83 Mbps/LUT.

Finally, our work was not able to carry out a fair comparison against other works since is the first to determine a high or low CQI in a FPGA hardware implementation using ML models, but it will be a reference for future similar works. In addition, it can be replicated in a hardware device such as an ASIC (Application-Specific Integrated Circuit) or FPGA (Field Programmable Gate Array).

## Figures and Tables

**Table 1:** Inputs and Outputs Attributes of the ML models and the Proposed CQI Label.

(a) Inputs and Outputs Attributes				
	**Attributes**			
Inputs	rsrp			
	rsrq			
	macStats_phr			
	macStats_totalBytesSdusDl			
	macStats_totalTbsUl			
	macStats_totalPduDl			
	macStats_totalPrbUl			
	macStats_totalPduUl			
	macStats_totalPrbDl			
	macStats_totalTbsDl			
	pdcpStats_pktRx			
	pdcpStats_pktRxSn			
	pdcpStats_pktTxSn			
	pdcpStats_pktRxBytes			
	pdcpStats_pktTxW			
Outputs	Low Quality			
	High Quality			
**(b) Channel Quality Indicator Defined by the 3rd Generation Partnership Project with the Proposed Label**				
**CQI**	**Label**	**Modulation**	**Code Rate × 1024**	**Efficiency**
0	Out of Range			
1	Low Quality	QPSK	78	0.1523
2		QPSK	120	0.2344
3		QPSK	193	0.3770
4		QPSK	308	0.6016
5		QPSK	449	0.8770
6		QPSK	602	1.1758
7	High Quality	16QAM	378	1.4766
8		16QAM	490	1.9141
9		16QAM	616	2.4063
10		64QAM	466	2.7305
11		64QAM	567	3.3223
12		64QAM	666	3.9023
13		64QAM	772	4.5234
14		64QAM	873	5.1152
15		64QAM	948	5.5547

**Table 2:** Hyper-parameters Used for the Developed Machine Learning Algorithms.

Algorithm	Considered Hyperparameters	Obtained Hyperparameters	Configuration
Decision Tree	criterion: {entropy, gini}, max_depth: {1:15}, min_samples_split: {2:4}, min_samples_leaf: {1:3}, min_weight_fraction_leaf: {0, 0.1, 0.01, 0.001}	criterion: {entropy}, max_depth: {6}, min_samples_split: {2}, min_samples_leaf: {3}, min_weight_fraction_leaf: {0}	Implemented Depths: five and six, Designs based on comparators
MLP	neurons: {1:10}, activation_function: {softmax, softplus, softsign, relu, tanh, sigmoid, hard_sigmoid, linear}, optimizer: {SGD, RMSprop, adagrad, adadelta}, batch_size: {10, 20, 40, 60, 80, 100}, learning_rate: {0.001, 0.01, 0.1, 0.2, 0.3}, weight_init: {uniform, lecun_uniform, normal, zero, glorot_normal, glorot_uniform, he_normal, he_uniform}, dropout: {0, 0.1, 0.2, 0.3, 0.4, 0.5, 0.6, 0.7, 0.8, 0.9}	neurons: {5}, activation: {relu}, optimizer: {RMSprop}, batch_size: {5}, learning_rate: {0.01}, weight_init: {glorot_normal}, dropout: {0}	MLP based on three dense layers, with the next configurations of neurons [5, 5, 1]
Logistic Regression	penalty: {l1, l2, elasticnet, none}, tol: {0.0001, 0.001, 0.01, 0.1}, C: { 1 × 10${}^{-4}$, 2.63 × 10${}^{-4}$, 6.95 × 10${}^{-4}$, 1.83 × 10${}^{-3}$, 4.83 × 10${}^{-3}$, 1.27 × 10${}^{-2}$, 3.35 × 10${}^{-2}$, 8.85 × 10${}^{-2}$, 0.23, 0.61, 1.62, 4.28, 11.28, 29.76, 78.47, 206.91, 545.55, 1438.44, 3792.69, 10000}, solver: {newton_cg, lbfgs, liblinear, sag, saga}, max_iteration: {10, 100, 1000}	penalty: {l2}, tol: {0.0001}, C: {10000}, solver: {newton_cg}, max_iteration: {1000}	Logistic Regression model based on the modules: Multiplier - Adder 1, Sigmoid, and Comparator
SVM	kernel: {poly, rbf, sigmoid, linear}, C: {1 × 10${}^{-2}$, 5.99 × 10${}^{-2}$, 0.35, 2.15, 12.91, 77.42, 464.15, 1.66 × 10${}^{4}$, 1 × 10${}^{5}$}, gamma: {0.001, 0.01, 0.1, 1, 10}	kernel: {rbf}, C: {464.1588}, gamma: {scale}	SVM model based on the modules Multiplier-Adder 1 and Comparators

**Table 3:** Resource Comparison of the Implemented Machine Learning Algorithms.

Machine Learning Technique	Latency	LUT	FF	DSP	Minimum Period (ns)	Max. Frequency (MHz)	Throughput (Mbps)	Efficiency (Mbps/LUT)	Balanced Accuracy	Sensitivity	Specificity
Decision Tree (depth 6)	25	890	1389	0	3.125	320	2355.20	2.6462	95.2626	94.88	95.64
Decision Tree (depth 5)	23	320	812	0	3.093	323.3107	3148.7650	9.8398	95.0180	94.12	96.91
Multi-Layer Perceptron	317	36,825	79,465	439	6.081	164.44	249.004	0.0067	94.0295	97.48	90.57
Logistic Regression	21	5886	1986	100	31.427	31.8197	727.3090	0.1235	93.0713	93.14	92.99
Support Vector Machine	25	4865	1922	75	16.993	58.8477	1129.8770	0.2322	92.0657	92.99	91.13

**Table 4:** Resource Comparison Among Different Works.

Work	Algorithm	Evaluation	Platform	Latency	LUT	FF	BRAM	DSP	Freq. (MHz)	Throughput (M)	Efficiency (Mbps/LUT)	Objective
This Work	Decision Tree (5) Decision Tree (6) MLP Logistic Reg. SVM	95.01% 95.26% 94.02% 93.07% 92.06%	Virtex-7	23 CC 25 CC 317 CC 21 CC 25 CC	320 890 36,825 5886 4865	812 1389 79,465 1986 1922	0 0 0 0 0	0 0 439 100 75	323.3107 320 164.44 31.8197 58.8477	3148.7 b/s 2355.2 b/s 249 b/s 727.3 b/s 1129.8 b/s	9.83 2.64 0.006 0.12 0.23	Low and High Channel Quality Classification
Lin Z. et al. [25]	Decision Tree	Acc: 89.10% 76.16% 76.26% 71.02% 39%	VCU1525	0.36 ms 0.26 ms 0.42 ms 3.97 ms 0.93 ms	63,079 73,800 54,198 169,334 59,401	- - - - -	486 480 384 1883 986	202 184 138 1126 191	308 305 300 170 266	- - - - -	- - - - -	Online Learning for Large Datasets
Choudhury et al. [26]	TM Decision Tree	Acc: ±68% ±88% ±70% ±85% ±90%	Virtex Ultrascale+	24 ms	894,000	6488	228	200	62	-	-	Dynamic Training for Binary Datasets
Novickis R. et al. [27]	FFNNs	MAE 0.0232 0.0232 0.0189	Zynq-7000 SoC ZC702	10.22 us 8.06 us 8.05 us	8958 8881 9607	- - -	8 8 10	14 20 20	100 100 100	1.52 Sam/s 2.02 Sam/s 2.02 Sam/s	- - -	Estimating Forces of an Electric Car
Kachris C. et al. [28]	Logistic Regression	Accuracy: 90%	Zynq FPGA SoC.	-	44177	47841	42	160	667	-	-	Framework for Data Analytic
Batista G. et al. [29]	SVM	Accuracy: 98%	Cyclone IV EP4CE115F29C7	1.648 us	1314	-	-	-	50	1.64 MOPS	-	Speech Recognition
Wu R. et al. [30]	SVM	Effective Utilization Rate: 94.82%	Xilinx ZYNQ 7Z020	274.50–40632 ns	12090	-	8	-	100	-	-	Matrix Computing with Changeable Dimensions

## Abbreviations

The following abbreviations are used in this manuscript:

DVN	Software-defined vehicular network
VANET	Vehicular ad hoc network
SDN	Software-defined networking
V2V	Vehicle-to-vehicle
V2I	Vehicle-to-Infrastructure
V2X	Vehicle-to-all
V2P	Vehicle-to-pedestrian
V2H	Vehicle-to-home
V2B	Vehicle-to-building
V2C	Vehicle-to-cloud
V2G	Vehicle-to-grid QoS
Quality of service	
CQI	Network Channel Quality Indicator
ML	Machine learning
GPU	Graphics processing units
FPGA	Field Programmable Gate Arrays
ASICs	Application-specific Integrated Circuits
MLP	Multi-layer perceptron
SVM	Support Vector Machine
FFNN	Feed-Forward Neural Network
PDR	Packet delivery ratio
IA	Artificial Intelligence
CNN	Convolutional Neural Network
LUT	LookUp Table
DSP	Digital Signal Processing
FF	Flip flop
BRAM	Block Random Access Memory
